# Acute intermittent hypoxia in neonatal rodent central nervous system facilitates respiratory frequency through the recruitment of hypothalamic areas

**DOI:** 10.1113/EP092303

**Published:** 2025-03-13

**Authors:** Rosamaria Apicella, Graciela L. Mazzone, Giuliano Taccola

**Affiliations:** ^1^ Neuroscience Department International School for Advanced Studies (SISSA) Trieste Italy; ^2^ Applied Neurophysiology and Neuropharmacology Lab Istituto di Medicina Fisica e Riabilitazione (IMFR) Udine Italy; ^3^ Instituto de Investigaciones en Medicina Traslacional (IIMT) CONICET‐Universidad Austral Pilar Buenos Aires Argentina; ^4^ Facultad de Ciencias Biomédicas Universidad Austral Pilar Buenos Aires Argentina

**Keywords:** c‐Fos, fictive respiration, hypothalamus, isolated central nervous system, partial oxygen pressure

## Abstract

Moderate and acute intermittent hypoxia (IH) facilitates respiration in adults, mostly by recruiting peripheral chemo‐/baroreceptors. As central chemoreceptors are widely expressed in immature brains, we hypothesized that IH modulates respiration at birth through a purely neurogenic mechanism involving the hypothalamus. The central nervous system (CNS) isolated from 0‐ to 3‐day‐old rats was perfused with four to eight brief (5 min) bouts of mild‐hypoxic/normocapnic modified Krebs solution, intermingled with 5‐min normoxic episodes, during continuous electrophysiological recordings from upper cervical ventral roots. An IH protocol did not modify bath pH, but superficial ventrolateral medulla and hypothalamic areas experienced lowered oxygen tension, more severe after the second postnatal day, with a partial recovery after each bout. Single exposures to mild hypoxia were well tolerated, and at birth often triggered a spontaneous epoch of irregular baseline activity (< 1 min) superimposed on respiratory events in both whole CNS preparations and spinal cords. Conversely, IH largely halted breathing activity after the second postnatal day, while at birth IH transiently increased the amplitude of respiratory bursts and stably sped up rhythm only when intact suprapontine structures were present. Rhythm acceleration was not directly correlated to instantaneous changes in tissue oxygen tension. After IH, respiratory frequency remained 260% higher than pre‐IH control for up to 60 min. Identical modulatory effects were observed with IH supplied through a HEPES buffer solution. Interestingly, IH increased electrical activity and cFos expression in hypothalamic areas without altering total cell number. These observations cast some light on the mechanisms of IH during development, with important insights about pediatric effects of repeated hypoxic episodes.

## Abstract (highlights)

- **What is the central question of this study?**
  Can IH modulate the respiratory rhythm already at birth, likely recruiting suprapontine structures?
- **What is the main finding and its importance?**
  Acute IH modulates respiratory rhythm frequency already at birth through a purely neurogenic mechanism that involves suprapontine brain centers. A distinct profile of modulation appears throughout the early postnatal development. An in vitro preparation of the entire central nervous system isolated from neonatal rats has been used to reveal that acute IH speeds up respiratory frequency with an increased activation of hypothalamic areas. The evidence provided casts some light on the mechanisms of IH during development, with important insights especially for the pediatric effects of repeated hypoxic episodes.

## 1 INTRODUCTION

A brief exposure to hypoxia is known to increase respiratory frequency in cats, an effect observed also after a decortication that kept the hypothalamus intact (Tenney & Ou, 1977). Similarly, in unanaesthetized adult rats, both intact and with a spinal cord injury (SCI), a single hypoxia bout speeded up respiratory frequency for up to 5 min, before returning to normoxia (Lee et al., 2017). An increased frequency of respiration has been observed also in the first 10 days after birth after a single episode of mild hypoxia (Julien et al., 2008).

However, repeated brief hypoxic episodes not only increased respiratory frequency (Sibigtroth & Mitchell, 2011), but also led to a long‐term facilitation (15–60 min) of the phrenic motor output (called phrenic long‐term facilitation, pLTF; Lee et al., 2017). It is likely that intermittent hypoxia (IH) involves two different neurotransmitter systems, as well as distinct intracellular signalling cascades (Devinney et al., 2013) that interact with each other through a powerful cross‐talk inhibition (Perim & Mitchell, 2019). The prevalence of one pathway over the other is determined by the magnitude of hypoxic events. On the one hand, severe chronic IH activates the sympathetic nervous system and triggers a systemic inflammation that leads to a reduced development of the whole organism and, in particular, a lower weight of the brain (Iturriaga et al., 2009; Nanduri et al., 2019; Serebrovskaya & Xi, 2015). On the other hand, moderate and acute IH (AIH) facilitate brainstem neural network activity through the activation of chemo‐ and baroreceptors contained in carotid and aortic bodies. Chemoreceptors use still‐unknown mechanisms to transduce values in the arterial blood – such as the reduced $P_{O_{2}}$ and pH, and the increased $P_{CO_{2}}$ – into neural signals that travel in the chemoafferent fibres that form the sinus branch of the glossopharingeal nerve (Iturriaga et al., 2021). Notably, pioneering repetitive electrical stimulation of the carotid sinus nerve, which conveys the activity of hypoxia‐sensitive carotid neurons to the brainstem, facilitates the respiratory output, demonstrating that pLTF relies on ‘afferent input from the carotid body (CB), rather than hypoxia of the brain’ (Millhorn et al., 1980). However, effects of AIH have been reported also in carotid denervated animals (Bavis & Mitchell, 2003; Martin‐Body et al., 1985; Sibigtroth & Mitchell, 2011). Indeed, in carotid denervated rats, AIH still promotes a residual form of pLTF. In this case, the effects of AIH have been mainly ascribed to variations in parasympathetic tone, leading to a systemic hypotension that exacerbates central nervous system (CNS) hypoxia (Perim et al., 2020; Sibigtroth & Mitchell, 2011). As opposed to the CB‐dependent pLTF following AIH, the denervation of carotid bodies unveils a residual form of pLTF that is directly linked to a reduced spinal oxygenation near phrenic motoneurons (Perim & Mitchell, 2019). In addition, after denervation of carotid bodies, loss of peripheral chemoreceptors is counterbalanced by additional central chemoreceptors, located in many loci of the brainstem and cerebellum, as well as diencephalon (Zoccal et al., 2023). In particular, neurons in the caudal hypothalamus that project to respiratory centres receive excitatory input during hypoxia (Horn & Waldrop, 1997).

Interestingly, while central chemoreceptors, also known as acid‐sensitive receptors, sense pH and $P_{CO_{2}}$ only (Wong‐Riley et al., 2013), certain caudal hypothalamic neurons may function as central oxygen chemoreceptors (Dillon & Waldrop, 1992; Horn & Waldrop, 1997; Horn & Waldrop, 2000). These latter central chemoreceptors are the main O_2_‐sensing mechanisms to contrast hypoxia at birth, when CB sensitivity to O_2_ is still low (Zoccal et al., 2023).

Although neurotransmitter systems and distinct signalling machinery are already expressed at birth, rat pups gradually express a respiratory long‐term facilitation (LTF) from the second day postnatal (Julien et al., 2008; McKay et al., 2004; Wong‐Riley et al., 2013). At birth, the chemosensitivity of CBs is transitorily silenced to compensate the sudden change in arterial $P_{O_{2}}$ when exiting the uterus. Afterwards, in the second neonatal week, anaesthetized rats with spontaneous breathing showed elevated amplitude and burst frequency during single hypoxic episodes (Reid & Solomon, 2014). Contrariwise, repeated hypoxic episodes protractedly increased the frequency of respiratory bursts throughout the AIH trial, and for up to 60–90 min after return to normoxic conditions (Julien et al., 2008; Reid & Solomon, 2014).

As the site that mediates the effects of AIH in newborns is still unknown, we wondered whether the facilitation of respiration in the first 2 days of life depends on the integrity of CBs and whether AIH‐induced modulation of respiratory frequency can still be observed in the absence of peripheral chemo‐baroreflexes. Moreover, to the best of our knowledge, it has never been reported whether hypothalamic neurons become activated during AIH at birth. However, we hypothesized that the facilitation of the respiratory frequency at birth relies on a purely neurogenic mechanism involving the hypothalamus, in line with the responses to hypoxia obtained in adult rats with denervated CBs (Horn & Waldrop, et al., 2000). To test this hypothesis, we set up an innovative experimental model of IH in vitro where the CNS was isolated from neonatal rats (Apicella & Taccola, 2023; Mohammadshirazi et al., 2023, 2025) and repetitively perfused with a mild hypoxic and normocapnic modified Krebs solution. Continuous electrophysiological recordings and selective staining with markers of neuronal activity demonstrated that moderate AIH stably potentiates the frequency of the respiratory rhythm with a distinct activation of the caudal hypothalamus.

These observations cast some light on the mechanisms of AIH during development, with important insights especially for the pediatric effects of repeated hypoxic episodes.

## 2 METHODS

### 2.1 Ethical approval

All procedures were approved by the International School for Advanced Studies (SISSA) ethics committee (ethics approval reference number 22DAB.N.52M) and are in accordance with the guidelines from the Italian Animal Welfare Act 24/3/2014 no. 26 implementing the European Union directive on animal experimentation (2010/63/ EU). The study complies with the ARRIVE guidelines and *Experimental Physiology*’s policies regarding animal experiments. All animals were provided by SISSA Vivarium and bred with free access to food and water, and under standard enriched housing regimes with wooden logs, cardboard tubes and food pellets in the cage. Every effort was made to minimize the number of animals used and to ensure their well‐being.

### 2.2 Surgery

In vitro experiments were performed on the entire isolated CNS with hindlimbs attached from 61 Wistar rats of either sex (0–3 days old; Mohammadshirazi et al., 2023). All pups were born in our animal care facility with an exact determination of birth date. Surgical anaesthesia was induced by hypothermia (−20°C, 8–9 min; Danneman & Mandrell, 1997; Goldberg, 2015; Phifer & Terry, 1986). After loss of pedal reflex, the rostral part of the head above the olfactory bulbs was ablated and animal was eviscerated. The preparation was then placed in a Sylgard‐filled dissection chamber covered with Krebs solution bubbled with 95% O_2_–5% CO_2_ at room temperature (21–25°C). Dissection continued under microscopic guidance (Apicella & Taccola, 2023). Briefly, after isolation of the whole brain from cranial bones, dorsal and ventral laminectomies were made down to the last thoracic segment (Th13) in order to keep intact lumbosacral vertebrae and all hindlimb connections. In 11 preparations, suprapontine structures were removed through surgical transection of brain tissue above superior colliculi (Okada et al., 1998; Voituron et al., 2005) to obtain a reduced preparation consisting of pons–medulla + spinal cord with legs attached. After a post‐dissection resting period of 30 min, all preparations were pinned down with the ventral surface facing upward in a recording chamber (5 mL volume) continuously superfused (flow rate 7 mL/min) with oxygenated (95% O_2_–5% CO_2_) Krebs solution containing (mM): 113 NaCl, 4.5 KCl, 1 MgCl_2_7H_2_O, 2 CaCl_2_, 1 NaH_2_PO_4_, 25 NaHCO_3_, and 30 glucose, pH 7.4. Temperature in the recording bath was progressively raised to 25–27°C and held steady during the experiment through a temperature controller (TC‐324C Warner Instruments, Hamden, CT, USA).

For hypoxic stimulation, a modified unoxygenated Krebs solution was prepared with an oxygen tension of ∼120 Torr (16% O_2_). Two IH protocols were created, consisting of either eight or four bouts of 5‐min perfusions with low‐oxygen Krebs solution intermingled by 5‐min reoxygenation phases in fully oxygenated Krebs solution (∼425 Torr). In six experiments, hypoxic solution was added with HEPES including (mM): 113 NaCl, 4.5 KCl, 1 MgCl_2_7H_2_O, 21 sodium isethionate, 10 HEPES, 1 CaCl_2_, 30 glucose, pH 7.4.

### 2.3 pH assessment

pH measurements in the recording chamber were obtained by placing a pH meter microprobe (resolution ±0.001, accuracy ±0.002; edge series, Hanna instruments, Woonsocket, RI, USA) in the bath at a depth of 2–3 cm. The probe bulb was kept stable in the solution for the entire duration of experiments.

### 2.4 Electrophysiological recordings

All recordings were taken after reaching a stable baseline (60–90 min from beginning of anaesthesia). Respiratory‐like motor activity was extracellularly recorded from either left (l) or right (r) cervical VRs using tight‐fitting monopolar glass suction electrodes filled with Krebs solution. DC‐coupled recordings were acquired with a differential amplifier (DP‐304, Warner Instruments; low‐pass filter = 10 kHz, high‐pass filter = 0.1 Hz, gain × 1000) and analog signals were further filtered by a noise eliminator (D400, Digitimer Ltd, Welwyn Garden City, UK). Signals were digitized at a sampling rate of 10 kHz (Digidata 1440, Molecular Devices, San Jose, CA, USA), visualized real time with the software Clampex 10.7 (Molecular Devices) and stored on a PC for offline analysis. For further calculations and figure editing, traces were processed offline using a digital low‐pass filter (10 Hz; Clampfit 10.4, Molecular Devices).

To trace the extracellular activity of hypothalamic areas, the recording electrode was placed laterally (mediolateral distance = 0.5 mm) in the right surface of the region between the pituitary stalk and the optic chiasma (bregma = −1.20 mm and lambda = 2.60 mm), then lowered perpendicularly in the tissue at a depth of 100–150 µm using a micrometric manipulator.

Field recordings were obtained using two different types of electrodes: a fine stainless steel electrode (FHC Inc., Bowdoin, ME, USA, impedance 11 MΩ at 1000 Hz) and a glass microelectrode (Harvard Apparatus, Holliston, MA, USA) filled with 3 M KCl (resistance 10 MΩ). AC‐coupled recordings were acquired with a differential amplifier (high‐pass filter = 300 Hz, gain × 1000) and digitized at a sampling rate of 250 kHz. Frequency of rhythmic burst hypothalamic areas was calculated as the occurrence of peaks in 5‐min bins, both in pre‐IH control and in the range 35–40 min after the end of the protocol.

### 2.5 Assessment of tissue oxygen content

Measurement of brain tissue $P_{O_{2}}$ was performed using an implantable fibre‐optic microsensor with a tip diameter of 50 µm (resolution 1.55 ± 0.08 Torr; accuracy ±1.54 Torr; OxyMicro System, World Precision Instruments, Sarasota, FL, USA). All $P_{O_{2}}$ measurements were temperature compensated (Zimmer et al., 2020). The microsensor was placed in a custom‐made glass housing and mounted on a micromanipulator to enable fine control in both vertical and horizontal planes. The tip was positioned on the ventral medullary surface, between the emergence of hypoglossal nerve rootlets and the midline, at a depth of 100 µm. Appropriate contact of the microsensor with the preparation surface was determined as the position where an abrupt change in the sensor output was observed (Okada et al., 1993). For oximetric assessments of hypothalamic areas, the probe tip was placed on the left surface of the region (mediolateral distance = 0.5 mm) between the pituitary stalk and the optic chiasma (bregma = −1.20 and lambda = 2.60 mm), then lowered perpendicularly in the tissue at a depth of 100–150 µm using a micrometric manipulator.

$P_{O_{2}}$ measurements were taken every second and were acquired directly through data acquisition software (OxyMicro Software, World Precision Instruments).

### 2.6 c‐Fos immunostaining and counting

After electrophysiological experiments, brains were fixed overnight with 4% paraformaldehyde at 4°C followed by tissue cryopreservation in 30% sucrose in distilled water, as described in Mohammadshirazi et al. (2023). Brains were cut into 20‐µm (coronal) sections using a sliding cryostat microtome. Sections, including hypothalamus, were selected according to bregma and lambda orientation using the coordinates provided by the rat brain atlas (bregma = −1.20 and lambda = 2.60 mm; Paxinos & Watson, 2006). Brain slices were incubated with blocking solution for 1 h and then with mouse monoclonal anti‐cFOS antibody (D‐1, sc‐8047, Santa Cruz Biotechnology, Inc., Heidelberg, Germany; 1:200) in 5% fetal bovine serum, 5% bovine serum albumin and 0.3% Triton X‐100 in phosphate‐buffered saline (PBS) overnight at 4°C. After three washes in PBS, floating sections were incubated for 2 h at room temperature with the appropriate goat anti‐mouse Alexa 488‐labeled secondary antibody (1:500, Thermo Fisher Scientific, Waltham, MA, USA). To visualize cell nuclei, slices were incubated in 1 µg/ml solution of 4′,6‐diamidino‐2‐phenylindole (DAPI). Sections were washed in PBS for 5 min, three times, and mounted using Vectashield mounting medium (Vector Laboratories, Burlingame, CA, USA) and coverslipped. The complete set of z‐stack hypothalamus images, typically to a depth of 4 µm, was obtained using confocal series Nis‐Eclipse microscope (Nikon, Amsterdam, Netherlands) with ×20 magnification. Optical sections were converted into three‐dimensional (3D) reconstructions and the number of cFOS positive nuclei were obtained using Volocity (https://www.volocity4d.com, Improvision, Coventry, UK) software.

### 2.7 Data analysis

Data analysis was performed using Clampfit 10.7 software (Molecular Devices) and data are expressed as means ± SD. Throughout the text, *n* is the number of preparations/animals used in each experimental group.

Each burst was defined as a period of sustained depolarization originating with a rapid onset from the baseline. Burst peak amplitude was considered as the vertical distance from the onset threshold (usually five times the standard deviation of baseline noise; Bracci et al., 1996). Bursting frequency was calculated using the formula *f* = 1/*T*, where period is calculated as burst number/time. Analysis of spontaneous baseline activity magnitude was obtained by the fast Fourier transform (FFT). Root mean square (RMS) of the resulting power spectrum was used to compare segments of baseline activity with the same duration. Statistical analyses were carried out with SigmaStat 3.5 software (Systat Software Inc, San Jose, CA, USA). Power spectra of hypothalamic field recordings were obtained using the FFT function. For each preparation, the peak of spectrum magnitude was explored in the frequency domain ranging from 0 to 3 Hz in pre‐IH control and in the last 10 min of the IH protocol.

All data in boxplots show the sample median (horizontal segment), the 75th and 25th percentiles (top and bottom edges of box), and 1.5 times the interquartile range (whiskers). All parametric values were processed using Student's *t*‐test (paired) to compare two groups of data, or with a one‐way repeated measures ANOVA for more than two groups. All multiple comparisons, either pairwise or versus a control group, were followed by *post hoc* test (Bonferroni's *t*‐test). Non‐parametric comparisons were performed using the Mann–Whitney rank sum test for two groups and, for more than two groups, Friedman's repeated measures ANOVA on ranks followed by Dunn's method for all pairwise and versus control multiple comparison procedures. Differences were considered statistically significant when *P* ≤ 0.050.

## 3 RESULTS

### 3.1 Neonatal CNS tolerates a single brief episode of mild hypoxia

**FIGURE 1:**
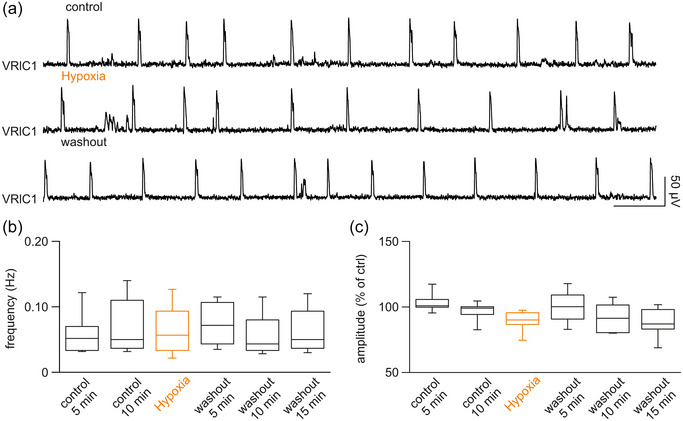
A single exposure to low‐oxygen medium does not alter fictive respiration. (a) Five‐minute traces of a spontaneous respiratory rhythm derived from the first cervical root on the left side of the spinal cord (VRlC1) in the whole CNS + legs attached preparation isolated from a PN 1 pup. Eleven respiratory events were recorded in fully oxygenated Krebs solution (top, control) and during perfusion with a low‐oxygen medium (middle, hypoxia) followed by the reoxygenation in control Krebs medium (bottom, washout). (b, c) Time courses for 5 min bins of mean frequency (b) and amplitude (c) pooled from 10 pups at PN 0/2. Orange bars correspond to the perfusion with low‐oxygen medium.

In the entire CNS isolated from a newborn pup (PN 1), a spontaneous respiratory rhythm recorded from an upper cervical VR (rC1) showed a mean frequency of 0.04F Hz with mean amplitude of single bursts of 69.78 µV. A following 5‐min perfusion using a physiological medium with a low‐oxygen content ($P_{O_{2}}$ = 120 Torr) did not change rhythmicity (0.04 Hz) or mean amplitude (65.13 µV) of respiratory events. The regularity of fictive respiration during hypoxic episodes (coefficient of variation (CV) period = 21.34%) is comparable to the one in control (CV period = 20.13%). Return to normoxic conditions showed an identical respiratory rhythm (CV period = 19.95%) with unaffected mean frequency (0.04 Hz) and mean burst amplitude (66.89 µV; Figure 1a).

Pooled data from 10 preparations obtained from 0‐ to 2‐day‐old rats indicate a stable time course as for mean frequency (Figure 1b) and amplitude (Figure 1c) throughout a brief bout of mild hypoxia.

Data suggested that fictive respiration in rats at birth is not affected by brief exposures to low‐oxygen solutions, confirming the greater resistance of neonatal tissue to mild hypoxic episodes compared to that of adults (Millar et al., 2017).

### 3.2 Serial brief applications of low‐oxygen medium intermittently drop tissue oxygen content

**FIGURE 2:**
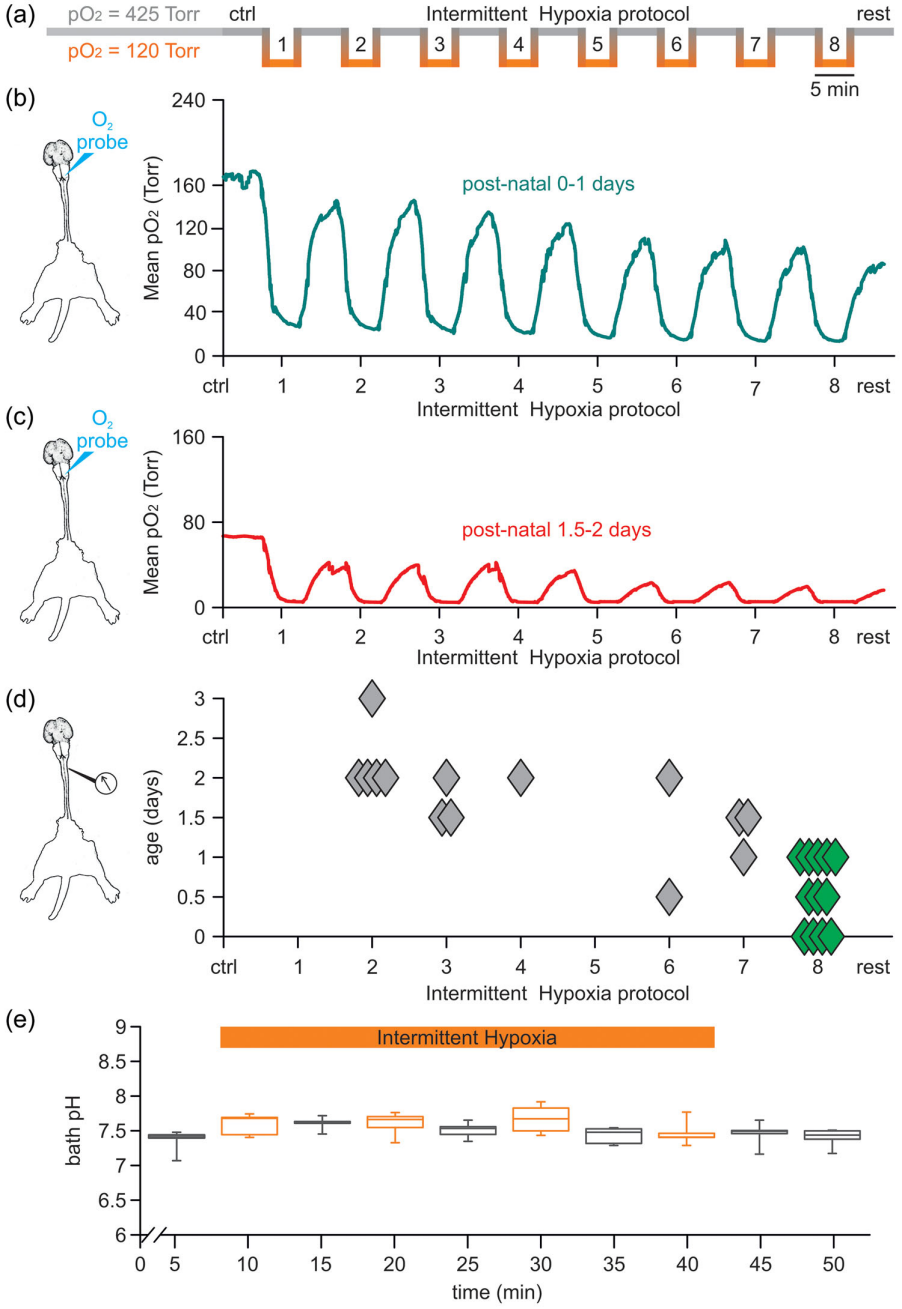
Intermittent hypoxia (IH) protocol with serial repetitions of brief episodes of low‐oxygen medium reduces tissue oxygen tension without affecting pH of the recording chamber. (a) Line schematizing the IH protocol, starting from a fully oxygenated Krebs solution ($P_{O_{2}}$ = 425) and composed of eight bouts of 5‐min perfusions with a low‐oxygenated Krebs solution ($P_{O_{2}}$ = 120) intermingled by 5‐min reoxygenation phases before returning to fully oxygenated conditions. (b) Oximetric assessments performed with a microprobe implanted in superficial layers of the ventrolateral medulla in the CNS + legs attached preparation (left cartoon) define the average time course of tissue oxygen levels throughout the entire protocol (green line) for four preparations from PN 0–1 animals. (c) The same setting as in (b) traces the continuous profile of the mean tissue oxygen content (red line) for three preparations from PN 1.5‐ to 2‐day‐old animals. (d) Respiratory rhythm recorded from upper cervical VRs (left cartoon) during the IH protocol. Each preparation (diamonds) is plotted against the age of the animal and the endpoint of fictive respiration that persists over all eight hypoxic repetitions only in preparations from younger animals (green diamonds). (e) Five‐minute bin time course for mean pH values of the perfusing solution in the recording bath throughout the four IH episodes (orange dots and bar) for five experiments.

As a single short bout of mild hypoxia did not change respiratory rhythmicity at birth, we wondered whether repetitive exposures to a transient episode of mild hypoxia would affect the respiratory output. Thus, the entire CNS isolated at birth (PN 0/2) was serially perfused with physiological solutions of low‐oxygen content (Figure 2a). In more detail, starting from a fully oxygenated solution with an oxygen content of about 425 Torr, $P_{O_{2}}$ in the medium was serially lowered to 120 Torr during eight phases of 5 min each, interspaced by normoxic episodes (wash) of equal duration (Figure 2a). The effect of this IH protocol on neural tissue was quantified using an oximetric probe implanted in the superficial layers of the ventrolateral surface of the medulla, where respiratory networks are located (Del Negro et al., 2009). Data from four rats at birth (PN 0/1) showed that perfusion with a fully oxygenated solution generates a mean $P_{O_{2}}$ on the tissue of 168 ± 21 Torr, eventually dropping to around 31 ± 14 Torr in the following perfusion with low‐oxygen medium (Figure 2b). The speed of changes in tissue oxygenation was equal for all repetitions (18 Torr/min). However, during washout with the fully oxygenated solution after each IH bout, tissue $P_{O_{2}}$ did not recover to baseline control values, but oxygen tension was progressively lowered, being 117 ± 39 Torr after the first four hypoxic episodes and 82 ± 43 Torr at the end of the eight repetitions (Figure 2b). When the same protocol of IH was applied to older animals (*n* = 3, 1.5‐ to 2‐day‐old postnatal pups), mean $P_{O_{2}}$ in control was 66 ± 26 Torr and consistently dropped to 6 ± 1 Torr throughout the following episodes. During full reoxygenations phases, $P_{O_{2}}$ was 30 ± 22 Torr in the middle of the protocol and 13 ± 7 Torr at the end of the protocol (Figure 2c). The functional meaning of the different $P_{O_{2}}$ levels when pups reached 2 postnatal days was tested as the continuous presence of a spontaneous respiratory rhythm from upper cervical VRs. Eight out of 11 preparations of 1.5‐ to 2‐day‐old pups stopped all breathing activities after the first episodes, while none withstood the seventh application nor recovered rhythm in the 5 min of washout in fully oxygenated solution (Figure 2d). Contrariwise, 12 PN 0/1 pups out of 14 lasted the entire protocol, keeping the respiratory rhythm throughout the following washout phase for up to 30 min after the last application of the Krebs hypoxic solution (green diamonds, Figure 2d). Since any manipulation of oxygenation of the perfusing solution did affect the final pH, especially when applied for a long time and using micro recording chambers (Carlin & Brownstone, 2006), we wanted to verify whether brief hypoxic repetitions change the mean pH of the solution in the large bath (total volume = 5 mL) we adopted to host the whole CNS in vitro with legs attached. Pooled data from five experiments showed a stable time course of the pH assessments continuously acquired during the IH protocol, with a mean value of 7.47 ± 0.18 pH units at the fourth hypoxic episode, similar to the values measured in both control (7.36 ± 0.16) and washout (7.46 ± 0.17, *P* = 0.072, one‐way repeated measures ANOVA; Figure 2e).

In summary, data collected demonstrate that our original IH protocol, which selectively perturbs the oxygen content without modifying the pH and consisting of serial brief applications of a low‐oxygenated Krebs medium, determines repetitive drops in medulla oxygen tension that do not fully recover to pre‐hypoxic values at the end of each bout. Interestingly, only pups at birth maintained a functional respiratory rhythm throughout the IH protocol while, after the second day of life, breathing activity was largely halted after a couple of hypoxic repetitions. This observation confirms that the second day of the first postnatal week is a pivotal milestone in the development of respiratory functions, as already reported (Wong‐Riley et al., 2013). For this reason, the following steps of the present study were restricted to preparations coming from newborns up to 24 h of life.

### 3.3 Mild IH facilitates frequency of the respiratory rhythm at birth through suprapontine centres

**FIGURE 3:**
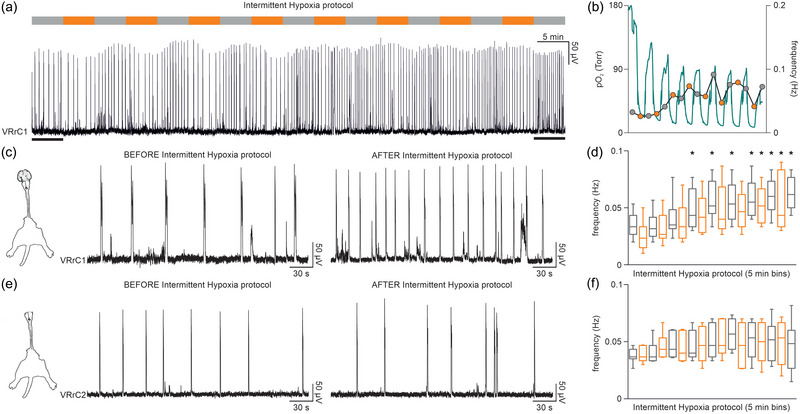
Intermittent hypoxia (IH) protocol increases respiratory pace only in the presence of suprapontine structures. (a) The IH protocol made of 8 serial 5 min‐long exposures to a low‐oxygen medium (upper bar) is applied to a preparation of whole CNS with legs attached isolated at birth. Respiratory rhythm is continuously recorded from VRrC1. (b) In another whole CNS + spinal cord from a PN 0.5 pup, the continuous profile of tissue oxygen levels (green trace) in the superficial layers of the ventromedulla is superimposed on the time course for 5‐min bins of the respiratory frequency, throughout the intermittent exposures to the hypoxic medium (orange dots). (c) Five‐minute traces from the same preparation of whole CNS with legs attached (left cartoon) in (a) display the respiratory rhythm underlined by the black bars, before (left trace) and after (right trace) the IH protocol. (d) Time course for 5‐min bins of mean rhythm frequency during the IH protocol (orange bars) in 12 whole CNS preparations with legs attached. Stars refer to the significant increase in respiratory pace. (e) Five‐minute traces from a brainstem (pons and medulla) + spinal cord with legs attached preparation isolated at birth (left cartoon), before (left trace) and after (right trace) the IH protocol. (f) Time course for 5‐min bins of mean rhythm frequency during the IH protocol (orange bars) in 10/11 preparations of brainstem (pons and medulla) + spinal cord with legs attached.

To explore whether repetitive intermittent changes in the tissue oxygenation of respiratory centres affect the breathing frequency at birth, spontaneous fictive respiration was recorded from the first cervical VR of the whole CNS in vitro with legs attached isolated from a PN 0 pup (Apicella & Taccola, 2023). In the fully oxygenated physiological solution used in control, spontaneous bursting progressively increased along the eight IH episodes of the protocol (Figure 3a). In another experiment performed on a PN 0.5 pup, the time course of the respiratory frequency showed a faster rhythm starting from the third IH bout, remaining higher throughout the following repetitions (Figure 3b). When superimposed on $P_{O_{2}}$ variations in the medulla, no direct correlation appeared between the instantaneous changes in tissue oxygen content and the speed of rhythm. At the end of all eight episodes of IH, respiratory frequency remained 260% higher than pre‐IH control (Figure 3c). This observation was repeated in 12 entire CNS preparations showing a significant increase in respiratory frequency after the third bout of hypoxia, then remaining significantly higher than control throughout the following reoxygenation phases and the last two hypoxic treatments until the end of the protocol (*P* ≤ 0.001, Friedman repeated measures ANOVA on ranks followed by multiple comparisons versus control group with Dunn's Method, *n* = 12, Figure 3d). Interestingly, the increased rhythmicity was monitored during the entire recording session, up to 30 min after the end of the protocol (data not shown). However, the same protocol applied to the sole brainstem (pons and medulla) + spinal cord with legs attached (Suzue, 1984) isolated from a pup of the same age (P0) did not change rhythm frequency (124%; Figure 3e). Pooled data from many experiments performed on reduced preparations deprived of suprapontine structures displayed no significant differences in the pace of fictive respiration during the entire IH, as well as at the end of the protocol upon return to the fully oxygenated solution (*P* = 0.266, one‐way ANOVA on ranks, *n* = 10/11, Figure 3f). In summary, a protocol consisting of brief mild hypoxic episodes intermittently repeated eight times facilitated the frequency of respiratory rhythm only when brain structures above the pons were kept intact. These results unveil the modulatory action of some suprapontine centres selectively activated by the repeated exposure to the hypoxic/reoxygenation phases.

### 3.4 Four episodes of mild IH modulate fictive respiration

**FIGURE 4:**
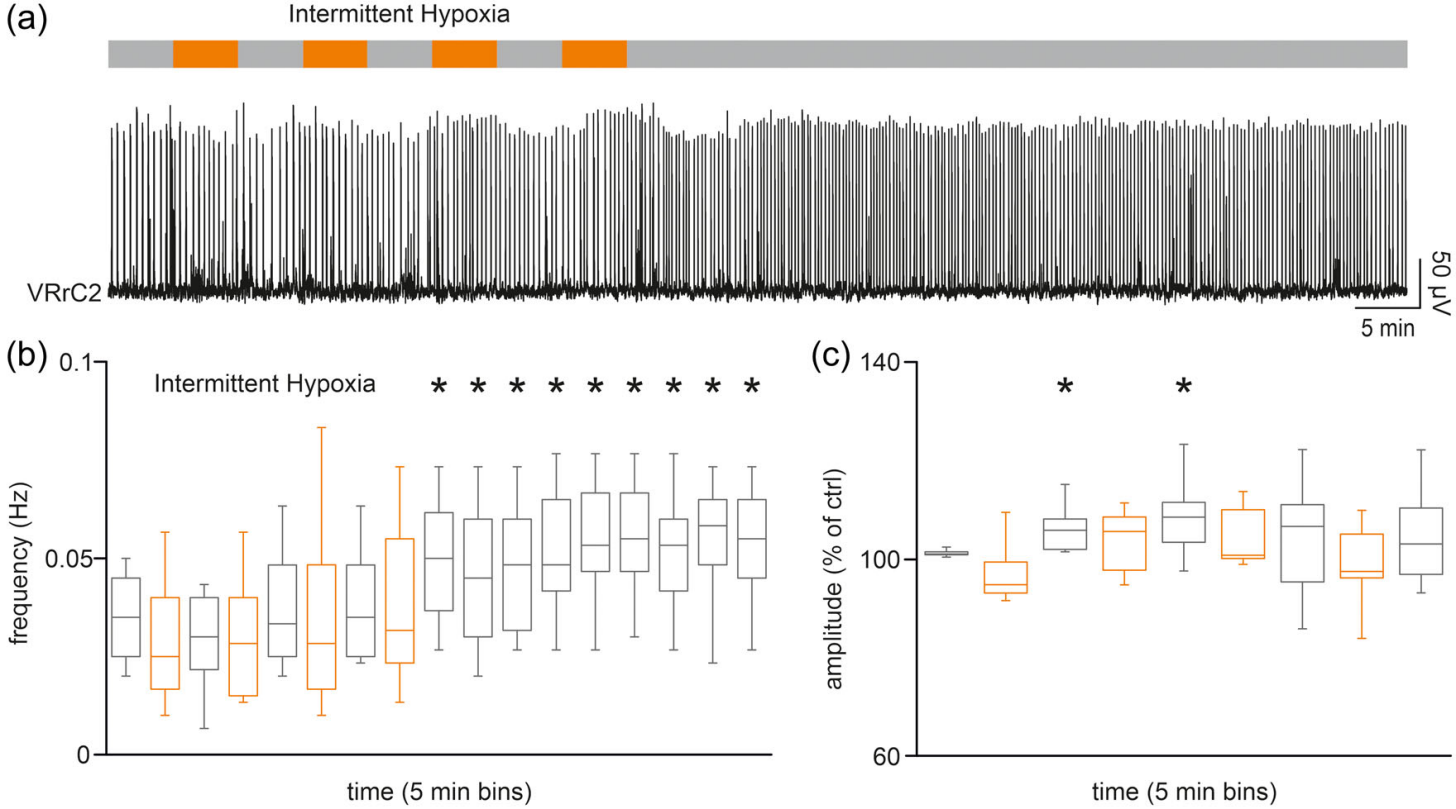
Four intermittent repetitions of the low‐oxygen medium are sufficient to speed up fictive respiration even after protocol termination, with higher burst amplitude only after the first hypoxic exposures. (a) The IH protocol made of 4 serial 5 min‐long exposures to low‐oxygen medium (upper bar) is applied to a whole CNS with legs attached preparation isolated at PN 1, during continuous recording of the respiratory rhythm from VRrC2. (b) Time course for 5‐min bins of mean rhythm frequency during the IH protocol (orange bars) in eight whole CNS with legs attached. Stars refer to the significant increase in respiratory pace lasting for up to 45 min after protocol ending. (c) Time course for 5‐min bins of mean burst amplitude during intermittent repetitions of the low‐oxygen medium (orange bars) in the same preparations as in (b). Stars highlight the significant increase in amplitude during the first two reoxygenation phases.

In the entire CNS in vitro, the IH protocol consisting of eight serial hypoxic episodes significantly speeded up frequency of the respiratory rhythm right after the first half of the protocol. Thus, we wondered whether a shorter IH protocol made of only four episodes might be enough to modulate the rhythm. A preparation of the entire CNS with legs attached taken from a PN 1 rat, showed a stable spontaneous fictive respiration of 0.04 Hz in control. The first two hypoxic episodes did not affect rhythmicity (100% and 100%), while the third (116%) and mainly the fourth (156%) episodes increased frequency, which remained stably higher than control for up to 50 min from the end of the IH protocol (163%; Figure 4a). Similarly, the mean amplitude of respiratory events transiently increased from 168.27 µV in control to 103% after the first IH, and even more during the third (112%) and fourth IH (109%) episodes. As opposed to frequency modulation, the IH protocol allowed amplitude to recover to the control values shortly after the end of the last hypoxic episode (101%, 10 min after the last IH). Of note, the ragged baseline in correspondence to the first three IH bouts refers to the appearance of irregular spontaneous discharges superimposed on the respiratory rhythm (Figure 4a).

The time course of the mean frequency calculated from eight animals (PN 0/1) displayed a statistical increase of the respiratory rhythm after the fourth application of hypoxic Krebs solution, lasting until 45 min from the end of IH (*P* = 0.003, one‐way repeated measures ANOVA followed by multiple comparisons versus control group with Bonferroni *t*‐test; Figure 4b). The same experimental sample was used to analyse the mean burst amplitude, revealing a transient statistical increase in the peak of respiratory events after the first and second IH applications (*P* ≤ 0.001, Friedman repeated measures ANOVA on ranks followed by multiple comparisons versus control group with Dunn's method, *n* = 8; Figure 4c). In summary, a shorter IH paradigm made of only four applications of hypoxic Krebs solution is sufficient to transiently increase the amplitude of respiratory bursts and to facilitate rhythm frequency, on a par with an IH protocol twice as long. Moreover, availability of a shorter IH protocol allowed us to explore the in vitro persistence of the facilitatory effect induced by IH on the respiratory rhythm, lasting for 45 min after the last mild hypoxic episode.

### 3.5 Four intermittent applications of a mild hypoxic buffer modulate fictive respiration

**FIGURE 5:**
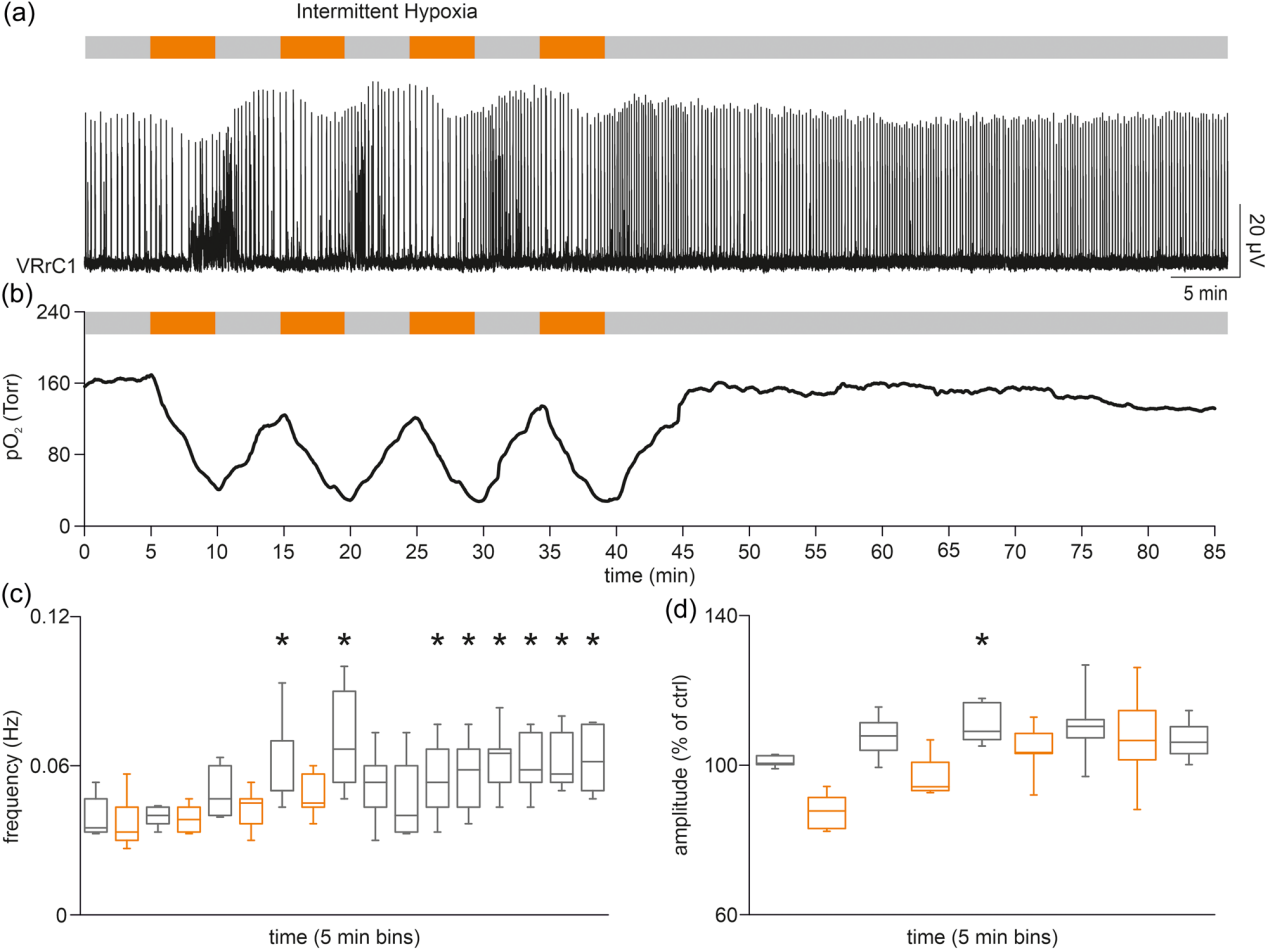
Four intermittent repetitions of low‐oxygen HEPES medium increase respiratory burst frequency and amplitude. (a) The IH protocol made of four intermittent exposures to a low‐oxygen Krebs solution buffered with HEPES at 7.42 pH units (upper bar) is applied to the CNS + legs attached preparation isolated at birth, during continuous acquisition of fictive respiration from VRrC1. (b) Continuous assessments of mean oxygen tension in superficial layers of the ventrolateral medulla during the IH protocol in HEPES (upper bar) for six preparations isolated from PN 0 to 1 pups. (c, d) Time courses for 5‐min bins of the mean frequency (c) and amplitude (d) for the same preparations in (b) Orange bars correspond to perfusion with low‐oxygen HEPES medium. Stars highlight the significant increase in frequency (c) and amplitude (d).

Since the manipulation of oxygen levels can change the medium pH (Carlin & Brownstone, 2006), in turn affecting the respiratory rhythm (Dubayle & Viala, 1998; Eugenín et al., 2006; Peever et al., 1999), we wanted to confirm that the respiratory modulation provided by our IH paradigm arises from serial changes in oxygen content and not from a slight alkalosis of the perfusing medium. Although real‐time pH assessments already showed no variations during the IH protocol (Figure 2e), to further exclude any potential influence of pH changes on respiratory activity, we adopted a HEPES physiological solution in which the pH was buffered to 7.44 ± 0.02 units. A stable fictive respiration of 0.04 Hz was derived from the entire CNS + legs attached preparation isolated from a PN 0 pup (Figure 5a). Application of serial bouts of mild IH in the presence of HEPES initially determined a small pace reduction (80%) soon followed by an augmented rhythmicity, corresponding to the third (225% than control) and fourth applications (225% than control; Figure 5a). Thereafter, breathing events remained accelerated (200% than control), even at the end of the whole experiment, 45 min after the last hypoxic HEPES perfusion (Figure 5a). Burst amplitude, which was 395.56 µV in control, increased after the first (116%) and second (117%) hypoxic episodes, with a progressive decay to baseline pre‐IH values starting from the end of the IH protocol (107%; Figure 5a). Interestingly, especially at the end of the first episodes, and less after the second and third, irregular spontaneous discharges made of fast and small episodes appeared superimposed on the respiratory rhythm (Figure 5a). Dynamics of the changes in tissue oxygenation on the superficial layers of the ventrolateral medulla were continuously traced and averaged for a total of six preparations from PN 0–1 pups (Figure 5b). A mean $P_{O_{2}}$ value of 163 ± 3 Torr in control is coherent with a mild hypoxic state. Afterwards, $P_{O_{2}}$ dropped to 84 ± 32 Torr at the end of the first hypoxic application of the HEPES solution, and then partially recovered to 93 ± 24 Torr during the subsequent reoxygenation phase (Figure 5b). The same trend was observed in the following three IH episodes. The $P_{O_{2}}$ value stabilized around 154 ± 3 Torr at the end of the IH protocol, albeit slightly decaying to 131 ± 1 Torr after 45 min of washout (Figure 5b). The same group of animals showed a time course for the mean rhythm frequency that indicated a significant increase in the fictive respiration pace after the third and fourth IH episode. Then, an even faster rhythmicity was recorded at the end of the protocol until 45 min after the last IH episode (*P* ≤ 0.001, Friedman repeated measures ANOVA on ranks followed by multiple comparisons versus control group with Dunn's method, *n* = 6; Figure 5c). On average, the modulatory effect on burst amplitude was only transient and reported after the second application of the hypoxic HEPES solution (*P* ≤ 0.001, Friedman repeated measures ANOVA on ranks followed by multiple comparisons versus control group with Dunn's method, *n* = 6; Figure 5d). Overall, an IH protocol made of four applications of a low‐oxygenated HEPES solution long‐lastingly speeded up the respiratory rhythm starting from the end of the protocol. Contrarywise, bursts of higher amplitude were recorded in the middle of the paradigm only. These results perfectly replicate the same modulatory effects of IH on fictive respiration that we reported above, using intermittent applications of a low‐oxygen Krebs solution.

### 3.6 A single hypoxic exposure triggers an episode of erratic baseline activity superimposed on the respiratory rhythm

**FIGURE 6:**
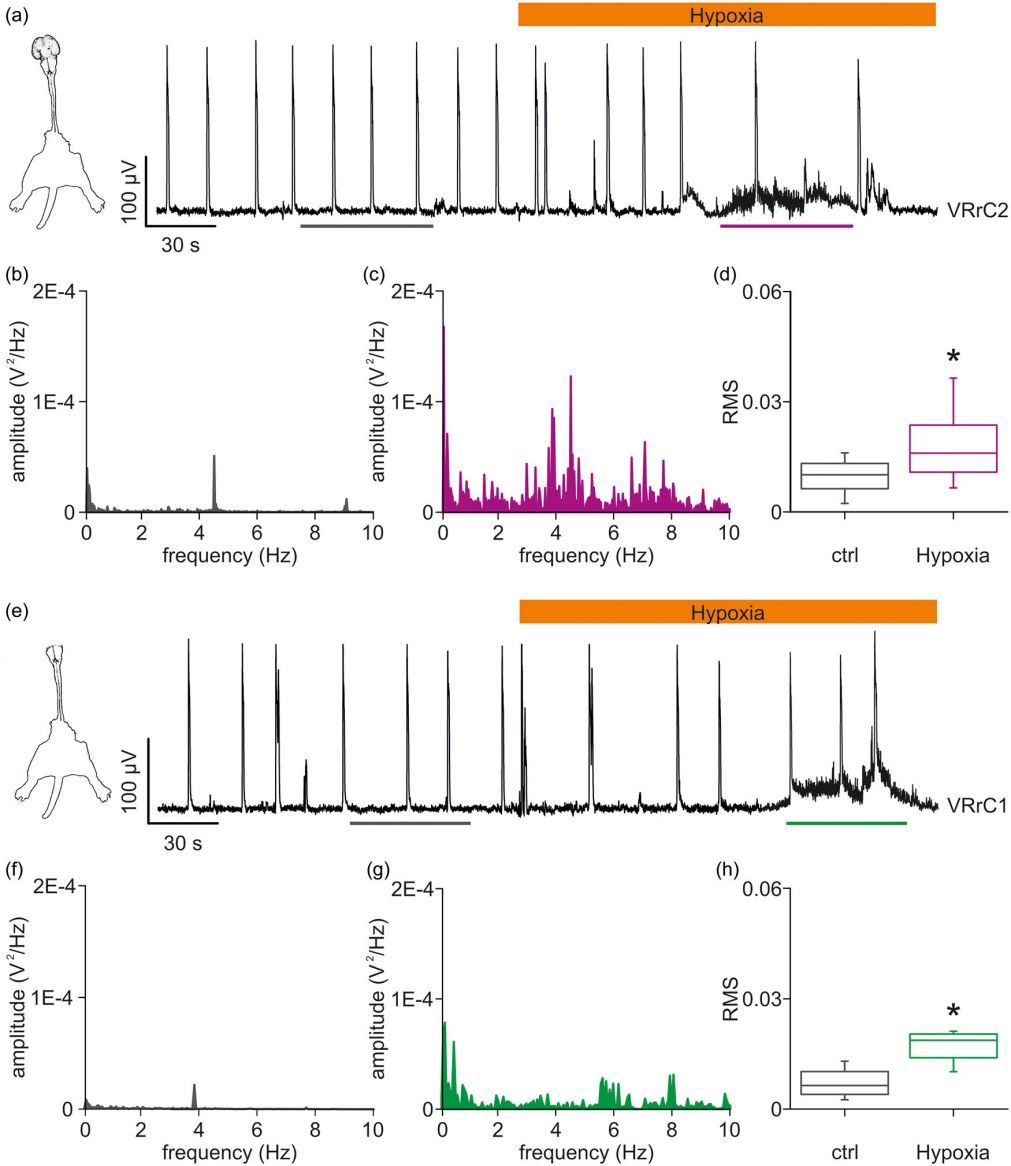
A first exposure to moderate hypoxia produces a transient increase in baseline motor pool activity regardless of the presence of suprapontine structures. (a) In a preparation of the entire CNS with legs attached (left cartoon) isolated at birth, fictive respiration is recorded from VRrC2 in control and for the first 150 s of mild hypoxia (orange bar). A transient delay of the respiratory rhythm corresponds to a small depolarization of the baseline, superimposed on a 56 s‐long episode of irregular activity (purple bottom bar). (b, c) For the same signal as in (a), equal length traces of baseline activity (grey bar in (a)) and early response to hypoxia (purple bottom bar in (a)) are analysed to define the power spectra in control (b) and at the beginning of perfusion with low‐oxygen medium (c). Note that occasional respiratory bursts occurring in the intervals considered were removed from spectral analysis. (d) Average RMS of power spectra from 18 preparations of the entire CNS with legs attached indicates the significant increase in the magnitude of spectra during brief episodes of erratic activity in response to hypoxia. (e) In the brainstem (pons and medulla) + spinal cord preparation with legs attached (left cartoon) isolated from a PN1 pup, fictive respiration is recorded from VRrC1 in control and for the first 150 s of mild hypoxia (orange bar). A transient baseline depolarization is superimposed on a 48 s‐long episode of irregular activity (green bottom bar). (f, g) For the same signal as in (e), equal length traces of baseline activity (grey bar in (e)) and of early response to hypoxia (green bottom bar in (e)) are analysed to define the power spectra in control (f) and at the beginning of hypoxic medium perfusion (g). Note that occasional respiratory bursts in the interval considered are removed from spectral analysis. (h) Star indicates the significantly higher mean RMS value during brief episodes of erratic activity in response to hypoxia. Data are collected from four preparations of brainstem (pons and medulla) + spinal cord with legs attached.

To better explore the transient stochastic discharges seldom observed during the first IH episodes, we performed a spectral analysis of baseline activity. In a CNS + legs attached isolated from a 0‐day‐old rat, a stable respiratory rhythm of 0.06 Hz was recorded from the second cervical VR (Figure 6a). When a hypoxic Krebs solution was applied for 5 min, rhythmicity slowed down (66% of control), and smaller events appeared (Figure 6a). After 88 s of perfusion with a low‐oxygen Krebs solution, a 56 s‐long bout of background erratic activity thickened the baseline. Erratic baseline discharges did not prevent a respiratory event from occurring, with unchanged amplitude (Figure 6a). The magnitude of the erratic background activity was quantified by comparing power spectra obtained from the analysis of two equal length segments of baseline, excluding respiratory events, in control (Figure 6b) and during hypoxia (Figure 6c). While the baseline in control showed a main frequency component centred on 4.5 Hz (Figure 6b), the spontaneous episode of erratic activity during perfusion with low‐oxygen Krebs displayed a more populated frequency content of higher amplitude with a peak around 0.12 Hz (Figure 6c). A similar epoch of erratic activity was reported in 18 out of 38 entire CNS preparations (47%) from PN 0–1 pups, with a mean duration of 57 ± 18 s. Magnitudes of baseline activity were quantified as mean RMS before and during hypoxia (Figure 6d). The significant increase in the mean RMS during hypoxia supports the consistent appearance of episodes of erratic background activity during the first exposure to hypoxic solution (*P* ≤ 0.001, paired *t*‐test, *n* = 18; Figure 6d).

Since, in our experiments, the modulating effects of IH on fictive respiration were conditioned by the presence of the entire CNS, eventually disappearing after ablation of suprapontine centres, we wondered whether also the episodes of background discharges originated from distinct brain structures. To this aim, in a preparation of the brainstem (pons and medulla) + spinal cord with legs attached, isolated from a 1‐day‐old rat, a spontaneous respiratory rhythm was recorded at 0.05 Hz during perfusion with a fully oxygenated Krebs solution (Figure 6e). Then, after 119 s of perfusion with low‐oxygen Krebs solution, a 48 s‐long episode of irregular baseline activity appeared superimposed on respiratory events of smaller amplitude (Figure 6e). Spectral analysis of two equal length segments of baseline activity showed only a single main component around 4 Hz in control (Figure 6f), as opposed to the denser spectrum during perfusion with hypoxic Krebs medium (Figure 6g). In four out of 11 entire brainstem (pons and medulla) + spinal cord preparations (36%) coming from PN 0–1 pups, an irregular activity appeared with a mean episode duration of 37 ± 8 s. This observation was quantified as a significantly higher mean RMS during hypoxia (*P* = 0.023, paired *t*‐test, *n* = 4; Figure 6h). Collectively, a single perfusion with a hypoxic Krebs solution triggered a brief episode of irregular baseline activity that transiently appeared during a continuous respiratory rhythm in almost half of the preparations and regardless of the integrity of suprapontine brain structures.

### 3.7 Hypothalamic areas are selectively activated by IH at birth

**FIGURE 7:**
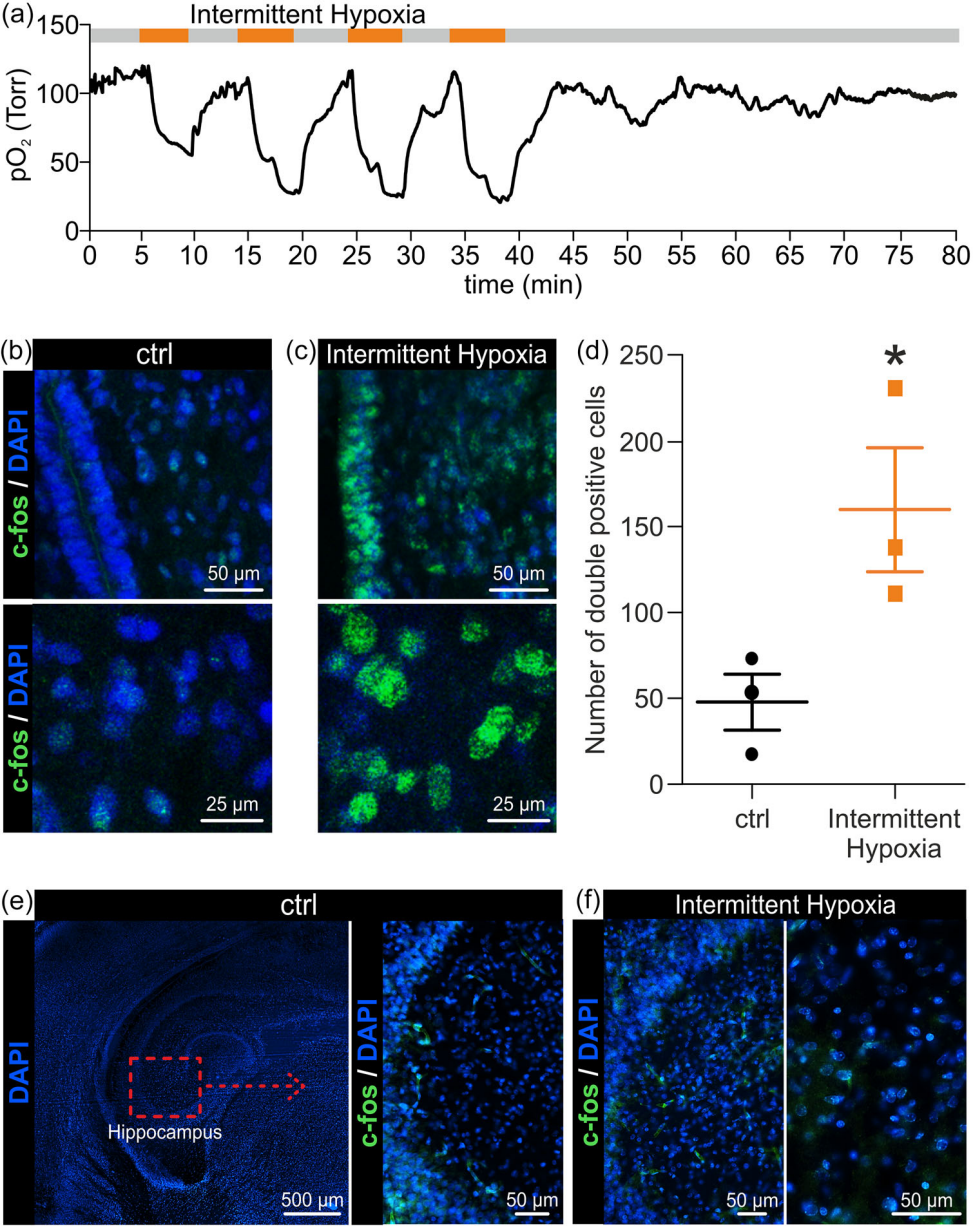
Intermittent hypoxia (IH) activates hypothalamic nuclei. (a) Mean time course of hypothalamus oxygenation during the IH protocol (*n* = 4). (b, c) Representative examples of double staining for the activity marker, cFos (green), and the cell nuclei label, DAPI (blue), of hypothalamic slices in control (b) and after four intermittent repetitions of a low‐oxygen medium (c) from two different preparations of the entire CNS with legs attached isolated at birth. (d) Whisker plots for the number of hypothalamic cFos‐positive nuclei in control and after IH. Each dot corresponds to a single preparation (three in control and three treated with IH) isolated from PN 0–1 pups. Star indicates the significant increase in the number of active cells in the hypothalamus after IH. (e, f) Double staining for DAPI (blue) and cFos (green) in the hippocampus for two different preparations in control (e) and after the IH protocol (f), respectively.

Among suprapontine structures, hypothalamic areas are involved in sensing tissue oxygen variations through central chemoreceptors (Dillon & Waldrop, 1992), as well as in controlling breathing frequency via direct pathways to respiratory networks located in the brainstem (Horn & Waldrop, 1997; Ryan & Waldrop, 1995). To explore whether hypothalamic areas are affected by our IH protocol, the oximetric probe was impaled in the hypothalamus and the time course of tissue $P_{O_{2}}$ was averaged among four CNS preparations. The average oxygenation of the hypothalamus in control (118 ± 32 Torr) was similar to that of the medulla. After a first drop in $P_{O_{2}}$ (61 ± 41 Torr), the following three hypoxic exposures further decreased hypothalamic $P_{O_{2}}$ to 26 ± 21 Torr, which then rapidly recovered to pre‐IH values after the end of each hypoxic episode (Figure 7a). Since oxygenation of the hypothalamus appeared modified during the IH protocol, we used the activity marker of neural cells, cFos, in hypothalamic slices obtained from whole CNS preparations isolated from PN 0–1 pups. When comparing two exemplar preparations, one untreated control and one subjected to four intermittent repetitions of low‐oxygen medium, DAPI staining of hypothalamic cell nuclei showed that the number of positive cells did not change after four episodes of IH (Figure 7b). However, while cFos immunohistochemistry visualized only a faint background co‐labelling in untreated animals in control (Figure 7b), a higher number of cFos positive cells was observed after IH treatment (Figure 7c). Pooled data from six animals (three untreated sham and three IH treated animals) showed that the number of hypothalamic cell nuclei did not change after IH (*P* = 0.697; *t*‐test; *n* = 3). However, a significant increase in cFos expression appeared in the hypothalamic areas of animals that underwent four intermittent applications of low‐oxygen Krebs solution (Figure 7d; *P* = 0.047; *t*‐test; *n* = 3). This evidence proves that in vitro IH paradigm activates hypothalamic areas, which likely modulate the pace of the respiratory rhythm at birth.

To demonstrate the causal link between cFos activation and the modulation of respiratory events during IH, we used two random preparations out of the ones adopted for cFos immunostaining of hypothalamic areas in control and after IH, respectively. Here, we analysed an adjacent non‐O_2_‐sensitive area, as the hippocampus, which revealed the absence of cFos expression after the IH protocol in both samples, confirming the selective activation of the hypothalamus during IH (Figure 7e,f).

### 3.8 Field recordings from the hypothalamus describe changes in neuronal activity during IH protocol

**FIGURE 8:**
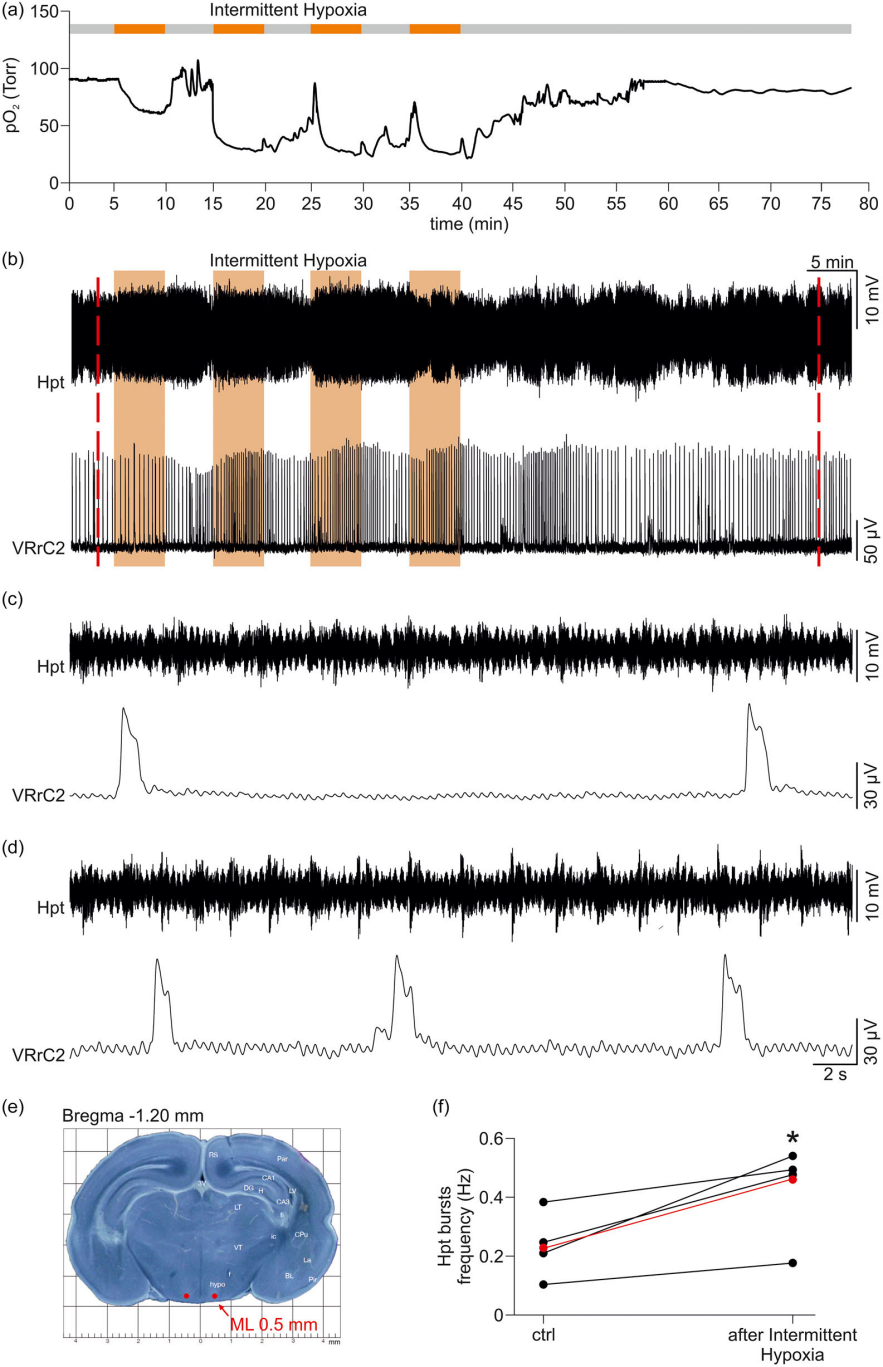
Intermittent hypoxia (IH) induces rhythmic hypothalamic discharges. Oxygenation profile of the hypothalamus (a), electrical activity from hypothalamic areas (Hpt, b, top), and fictive respiration from VRC2 (b, bottom) were simultaneously acquired in the same CNS preparation (P0) throughout the entire IH protocol. (c, d) Magnifications of traces in (b) (dashed red lines) illustrating oscillations from the hypothalamic areas (Hpt) and respiratory rhythm from VRC2, both in pre‐IH control (c) and 35 min after the end of IH protocol (d). Note the increased frequency for both hypothalamic areas (Hpt) and respiratory bursts after IH. (e) Electrophysiological electrodes and oxymetric probes are impaled on both sides of the hypothalamus at a depth of 100 µm from the surface, as indicated by the red dots in the atlas plane (bregma = −1.20 mm; lambda = 2.60 mm; mediolateral distance = 0.5 mm). (f) Plots and line graph for the frequency of hypothalamic (Hpt) oscillations, in pre‐IH control and from 35 to 40 min after the end of IH protocol (four repetitions). Red dots and line correspond to mean values (*n* = 4).

To better explore the link between the functional and immunohistochemical data referring to hypothalamic areas activated by the IH protocol, we performed four new experiments to acquire field recordings from the hypothalamus, paired with simultaneous cervical nerve recordings and oximetric assessments. In a sample experiment, the time course showed that hypothalamic oxygenation dropped from 90 Torr of pre‐IH control, to 60 Torr during the first perfusion with the hypoxic medium (Figure 8a). It is noteworthy that subsequent hypoxic episodes caused dramatic decreases in tissue oxygen (27 Torr for the 2nd, 25 Torr for the 3rd, and 26 Torr for the 4th) before recovering to baseline levels at the end of the IH protocol (88 Torr, 40 min after the end of IH). Simultaneous field recordings were performed by lowering electrodes into the hypothalamus at the same transverse plane used for cFos staining. Interestingly, no signals were acquired on the surface of the hypothalamus, while electrical signals appeared at a depth of 100–150 µm from the ventral surface (red dots, Figure 8b, top), which vanished when further lowering the tip of the electrode beyond 200 µm. In pre‐IH control, an oscillatory bursting rhythm appeared from the hypothalamus with a frequency of 0.21 Hz (Figure 8b,c, top), which was not related to the respiratory pace (0.04 Hz; Figure 8b,c, bottom). While the first hypoxic exposure did not change respiratory frequency, the following hypoxic episodes increased frequency and amplitude of respiratory bursts (Figure 8b, bottom). At the end of IH, rhythm remained accelerated for the entire duration of the recordings. From 35 to 40 min after the end of the protocol, the oscillatory pattern from hypothalamic areas was 0.54 Hz (Figure 8b,d, top) with a higher respiratory frequency (0.06 Hz; Figure 8b,d, bottom). Pooled data showed that the frequency of hypothalamic bursting after IH significantly increased by 88 ± 54% compared to pre‐IH control (Figure 8f, *P* = 0.050, paired *t*‐test, *n* = 4). This observation indicates a causal link connecting tissue oxygen dynamics in the hypothalamus, with hypothalamic activity, and fictive respiration during IH.

## 4 DISCUSSION

Here we present an essential in vitro model of intermittent mild hypoxia applied at birth through the repetitive superfusion of the entire CNS isolated from neonatal rats with a low‐oxygen medium, which does not affect pH. Although in the first 2 days of life, a single exposure to the low‐oxygen medium was well tolerated by the preparation and did not affect the respiratory rhythm, distinctive effects appeared when serial brief repetitions of mild hypoxic episodes were alternated with normoxic phases of equal duration. Indeed, a few epochs of IH lead to deterioration of fictive respiration until its complete suppression in animals older than 1 day of age. Conversely, neonates tolerated IH well up to eight brief episodes. The opposite effects observed in animals at birth and in 1‐day‐old pups reflect their own cumulative drop in tissue oxygen, which reflected a severe hypoxia in older pups and a moderate one in younger animals. At birth, application of at least four repetitions of intermittent mild hypoxia facilitated the amplitude of respiratory events during early hypoxic exposures, and increased rhythm frequency during and after the protocol. Any facilitation of the respiratory rhythm disappeared with the ablation of supraspinal centres, mirroring a higher activation of hypothalamic nuclei. However, regardless of the integrity of supraspinal structures, the first exposure to hypoxic media corresponded to an irregular high frequency activity of spinal motor pools, which appeared as a thicker baseline signal. A limitation of the current study is the random use of both sexes, while sex‐dependent differences in response to hypoxia are already present at birth (Netto et al., 2017).

### 4.1 Brief intermittent perfusion with low‐oxygen medium did not affect pH

In our IH model, we reduced sample oxygenation by turning off the carbogen mixture (O_2_ 95%, CO_2_ 5%) bubbling into the perfusing solution. Without the external supply of CO_2_, the non‐oxygenated solution remained in contact with environmental CO_2_ (almost 0.04%), which is far from the value of the carbogen mixture. Since the bicarbonate buffer requiring a constant supply of CO_2_ is also the main buffer system used for our perfusion medium, the solution pH may have been alkalinized by the transient interruption in the CO_2_ supply. In this regard, changes in the pH and $P_{O_{2}}$ of the solution in the recording chamber have been quantified upon the transitory stop of the perfusion (Carlin & Brownstone, 2006). In the study of Carlin & Brownstone (2006), the authors registered an increase of almost 0.4 pH units during the first 200 s, and a slow decay in oxygen tension, after stopping the supply of carbogen gas. Furthermore, they demonstrated that pH increased and $P_{O_{2}}$ reduced in an inverse relationship with the volume of the solution contained in the recording chamber (on average 0.5 mL). Notably, a rate of only 0.06 pH units per minute was reported for the largest volume considered (1.2 mL; Carlin & Brownstone, 2006). However, the CO_2_ value and the pH of the solution in the recording chamber do not directly parallel the pH of extracellular fluids, since tissue retains higher CO_2_ levels than the perfusing solution (Voipio & Kaila, 1993). Furthermore, during hypoxia, the concomitant decrease in $P_{O_{2}}$ leads to extracellular acidification, which partially compensates for the rise in pH due to CO_2_ deprivation (Hansen, 1985). Alkalinization modulates excitability of spinal neurons by affecting calcium currents mediated by voltage gated calcium conductances (Carlin & Brownstone, 2006). Additionally, alkalinization of the perfusing solution has been reported to slow down the respiratory rhythm in isolated preparations (Dubayle & Viala, 1998; Eugenín et al., 2006; McLean et al., 1995).

In our experiments, no changes in the pH of the solution in the chamber were reported when continuously measured during application of the non‐oxygenated solution deprived of the CO_2_ supply. Moreover, compared to the slices of neonatal spinal cord used in Carlin and Brownstone's study, our entire CNS in vitro required a larger recording chamber (5 mL) in which gases in the perfusing solution are slowly diluted when their supply is modified. Indeed, the pH of our preparation was not perturbed by the transitory reduction in CO_2_ during IH, as measured by the pH probe in the bath. As a matter of fact, we reported an increase in the frequency of the respiratory rhythm, excluding any alkalinization which would have reduced it (Dubayle & Viala, 1998; Eugenín et al., 2006; McLean et al., 1995). Eventually, IH performed in the presence of the HEPES buffer provided identical modulatory effects on fictive respiration, confirming that, in our study, facilitation of the respiratory rhythm by IH was not affected by any changes in pH.

### 4.2 IH provides a multilevel interaction with the respiratory neuronal system

In our study using animals right after birth, the instantaneous changes in tissue oxygen content assessed in the brainstem were not directly correlated to the speed of rhythm. This suggests that changes in the respiratory frequency might not come from a direct alteration of motor pool excitability induced by hypoxia. Contrariwise repetitive episodes of mild hypoxia increase breathing frequency, likely by modulating the mechanisms that control intrinsic rhythmicity (Del Negro et al., 2009). This effect is demonstrated in the entire CNS where at least four serial brief exposures to mild hypoxia generated an increased fictive respiration pace, which remained faster even after the end of the protocol. Prior to that, recordings from upper cervical spinal roots showed an amplitude of respiratory events that were transitorily augmented around half‐way through the protocol, reminiscent of the facilitation of polysynaptic pathways sustaining pLTF (Lee et al., 2017). Furthermore, at the beginning, a first exposure to the hypoxic medium elicited a brief episode of irregular activity from VRs regardless of CNS integrity. The different onset timing of these three effects and their distinct need of suprapontine structures suggest that IH in newborns may exploit different mechanisms and multiple cellular targets. Indeed, a direct chemosensitivity to low levels of oxygen is a quite common feature of neurons at birth. As a result, in brainstem slices, changes in oxygen supply modulate the firing activity of neonatal medullary neurons (Matott et al., 2014). Direct oxygen sensitivity of neurons relies on multiple classes of voltage‐dependent ion channels, which are in turn modulated by several O_2_‐dependent membrane mechanisms (López‐Barneo, 1996), including conformational changes of channel structure, activation of specific binding sites for second messengers and coupling with intracellular cascades (Lahiri et al., 2006). Distinct types of oxygen‐sensing channels might be involved, depending on the different district of the CNS. For instance, in inspiratory neurons, L‐type Ca^2+^ channels contribute to the early hypoxic response (Mironov & Richter, 1998), while in the neocortex and the substantia nigra, K^+^ channels are modulated during oxygen deprivation (Jiang & Haddad, 1994). Furthermore, hypoglossal motoneurons express ‘leak’ K^+^ TASK channels (Sirois et al., 2002; Talley et al., 2000), which confer on them oxygen‐sensing properties (Buckler et al., 2000). Hence, although yet unreported, also spinal respiratory motoneurons may own an intrinsic sensitivity to hypoxic conditions, and further studies using our protocol on cultured spinal motoneurons could elucidate this point. Overall, the IH protocol introduced in the present study affected the neonatal respiratory neural system at multiple levels, revealing the possibility to simultaneously trace both network‐based effects and direct excitation of motor pools.

### 4.3 Irregular nerve discharges appear at the first exposure to hypoxic medium

In the present study, we report a transient increase in non‐respiratory activity from cervical VRs a couple of minutes after the first exposure to the hypoxic medium. This effect was seldom replicated during the following hypoxic sessions of the protocol and did not require the presence of suprapontine structures, since the same short episode of irregular activity appeared also in preparations of the sole brainstem (pons + medulla) and spinal cord. Acute hypoxia directly facilitates the oligosynaptic component of spinal reflexes recorded from cervical motor pools (Eccles et al., 1966) also in spinal cords isolated from newborns, unless GABAergic and glycinergic synaptic transmission is pharmacologically blocked (Ataka et al., 1996).

We speculate that the transient irregular activity appearing in our neonatal preparation at the first exposure to hypoxia might be mediated by an enhanced inhibitory transmission mediated by GABA and glycine. In neonates, the transient facilitation of inhibitory transmitters induced by hypoxia might represent a protective mechanism to contrast hypoxia‐induced hyperexcitability (Haddad & Donnelly, 1990) and to prevent the cellular damage resulting from an acute lack of oxygen. Interestingly, the application of GABA and glycine to isolated spinal cords from neonatal rats elicits random episodes of irregular discharges from VRs (Rozzo et al., 1999), acknowledging the depolarizing nature of chloride conductance in the immature CNS, when chloride accumulates in neurons.

Consequently, glycine and GABA depolarize immature spinal neurons and generate a net outflux of bicarbonate ions (Hübner & Holthoff, 2013) that contributes to the intracellular mild acidosis that protects neonatal preparations against hypoxia (Zhu et al., 2019). This evidence might account for the greater tolerance to hypoxia that neonatal tissues (P0–P1) displayed in the current study, as well as in literature (Haddad & Donnelly, 1990; Krnjević, 1999; Luhmann et al., 1993).

From the second week postpartum, adenosine antagonists reduce the early component of spinal reflexes a few minutes after stopping oxygenation (Lloyd et al., 1988), mirroring the crucial role played by adenosine in adults to modulate the respiratory rhythm especially after IH (Perim et al., 2020). Indeed, at this developmental stage, the extracellular level of adenosine increases after acute hypoxia due to its release from astrocytes and intracellular ATP depletion (Takahashi et al., 2010). However, adenosine release is highly susceptible to age. Indeed, hypoxic‐induced adenosine release in 0‐ to 3‐day‐old rats was negligible when measured by selective enzymatic biosensors (Otsuguro et al., 2011). This evidence excludes the involvement of adenosine in the respiratory effects of moderate IH in the early days of life, as further explored herein (Perim et al., 2020). However, an alternative pathway may be involved in the persistent increase in phrenic nerve activity without requiring adenosine accumulation, as it exploits the activation of G_q_ protein‐coupled metabotropic receptors, new brain‐derived neurotrophic factor protein synthesis and protein kinase signalling within phrenic motoneurons (Devinney et al., 2013; Perim & Mitchell, 2019).

### 4.4 Hypoxia recruits hypothalamic areas

The hypothalamus contains neuronal nuclei crucial for the control of cardiorespiratory functions (Dampney, 2015) with direct projections to respiratory centres in the brainstem, such as the periaqueductal grey area (Vertes & Crane, 1996). In addition, the posterior hypothalamus contains neurons intrinsically sensitive to hypoxia, as they can be activated by hypoxia to increase respiration (Neubauer & Sunderram, 2004). Functionally, hypothalamic neurons are selectively excited by brief hypoxic episodes resulting in a pronounced increase of respiratory frequency (Dillon & Waldrop, 1993), which is eventually reduced by the pharmacological blockade of synaptic transmission in the caudal hypothalamus (Dillon & Waldrop, 1992). Similarly, the present study demonstrates that the repetitive bouts of mild hypoxia that speed up the respiratory rhythm also activate hypothalamic nuclei already at birth. The successful recordings from an isolated CNS preparation demonstrate the purely neurogenic source of hypothalamic activation through IH, which does not require the involvement of peripheral chemoreceptors. This evidence strengthens previous in vivo observations about the increased firing frequency of hypothalamic neurons during hypoxia, after vagus denervation (Dillon & Waldrop, 1993). However, the present study does not elucidate whether hypothalamic areas are directly activated by IH or if their recruitment is secondary to other brain areas. Notably, in neurons dissociated from the caudal hypothalamus, both rapidly inactivating and persistent Na^+^ currents are significantly enhanced by a brief hypoxic stimulus and lead to cell depolarization and increased firing frequency (Hammarström & Gage, 2000; Horn & Waldrop, 2000). Moreover, oxygen‐sensing neurons exploit the direct sensitivity of L‐type Ca^2+^ channels to change in the level of oxygen, as Ca^2+^ currents are being activated by hypoxia (Neubauer & Sunderram, 2004). In the hypothalamus, L‐type Ca^2+^ currents account for about one‐third of the total voltage activated Ca^2+^ currents (Ishibashi et al., 1995) and they mediate multiple modulatory actions in hypothalamic areas, such as the regulation of circadian rhythms (Kononenko et al., 2008), the control of food intake (Ma et al., 2007) and the release of hormones by neuropeptides (Avelino‐Cruz et al., 2009; Chen & Xu, 2003; Van Goor et al., 2000). Plasticity of L‐type Ca^2+^ channels and their upregulation in the hypothalamus contribute to activity‐dependent events, such as morphine dependence (Ramkumar & el‐Fakahany, 1988) and the patterned release of hormones (Quinlan et al., 2008). We speculate that repetitive stimulation with IH might serially increase Ca^2+^ concentration in hypothalamic neurons to reach the threshold for triggering Ca^2+^‐dependent plastic events that facilitate respiratory network activity in the mid and long term. In addition, the tissue oxygen profile of the hypothalamus indicates that a first brief episode of hypoxia causes a higher $P_{O_{2}}$ than the three following hypoxic repetitions, and it may be suggested that repetitive brief exposures to mild hypoxia override the intrinsic compensatory systems triggered by an acute reduction in tissue oxygen (Kemp, 2006; López‐Barneo et al., 2010). These O_2_‐sensing properties of hypothalamic neurons along with changes in tissue oxygenation during IH might explain the distinct cFos activation found in our study, as well as the lack of respiratory modulation by IH after ablation of suprapontine centres. Finally, albeit more scattered due to the mixed nature of cumulative input converging onto hypothalamic areas, field recordings during IH define a causal link among the modified hypothalamic activity, the increased burst frequency and amplitude of the respiratory rhythm, and the tissue oxygen dynamics in the hypothalamus.

### 4.5 Acute IH in neonatology

The repeated brief hypoxic episodes that lead to pLTF (Lee et al., 2017) also support the creation and adoption of groups of protocols that combine serial brief episodes of hypoxia intermingled by normoxic exposures (acute IH, AIH) to selectively improve the magnitude of respiratory output. This effect has not been reported though with continuous hypoxia (9 or 20 min). Moving from animal studies, AIH‐induced pLTF has been successfully introduced as a therapeutic intervention for restoring respiratory and motor functions in adult subjects with SCI or other neuromotor disturbances (Vose et al., 2022). A therapeutic AIH protocol has been defined as composed of three to five episodes of moderate isocapnic hypoxia ($P_{O_{2}}$ > 35 mmHg) lasting 2–5 min each, separated by 5‐min intervals (Vose et al., 2022). Albeit after over 20 years of clinical research, translation of AIH is still burdensome, as almost 40% of SCI participants have been considered minimal responders (Vose et al., 2022).

Well‐controlled, moderate AIH has also been proposed as a potential therapeutic tool to treat young children against a variety of pathological conditions, such as bronchial asthma, allergic and autoimmune disorders, cerebral palsy, and obesity (Serebrovskaya & Xi, 2015). However, handling IH as a pediatric clinical intervention is much more challenging for obvious ethical concerns and several pathophysiological reasons. For instance, in newborns, small differences in the dosage of IH might trigger adaptive as well as maladaptive plasticity. Episodes of severe chronic IH sustain autonomic and cardiorespiratory alterations that increase the likelihood of developing childhood cardiovascular diseases, cognitive and behavioural disturbances, as well as psychiatric disorders. These results show the urgency for further basic studies, which can potentially benefit also neonates, to exploit current knowledge on AIH through a bidirectional path that proceeds also from the bedside back to the bench (Vose et al., 2022).

## AUTHOR CONTRIBUTIONS

Giuliano Taccola and Rosamaria Apicella contributed to the study of conception and design. Giuliano Taccola, Rosamaria Apicella, and Graciela L. Mazzone performed acquisition and analysis of data for the work. The manuscript was drafted and critically commented by all authors. All authors have read and approved the final version of this manuscript and agree to be accountable for all aspects of the work in ensuring that questions related to the accuracy or integrity of any part of the work are appropriately investigated and resolved. All persons designated as authors qualify for authorship, and all those who qualify for authorship are listed.

## CONFLICT OF INTEREST

None declared.

## Data Availability

The datasets generated during and/or analysed during the current study are available from the corresponding author on reasonable request.
